# Blastocyst-stage embryos provide better frozen-thawed embryo transfer outcomes for young patients with previous fresh embryo transfer failure

**DOI:** 10.18632/aging.103055

**Published:** 2020-04-15

**Authors:** Lanlan Fang, Jingyan He, Yang Yan, Qiongqiong Jia, Yiping Yu, Ruizhe Zhang, Jung-Chien Cheng, Ying-Pu Sun

**Affiliations:** 1 Center for Reproductive Medicine, Henan Key Laboratory of Reproduction and Genetics, The First Affiliated Hospital of Zhengzhou University, Zhengzhou, China

**Keywords:** frozen-thawed embryo transfer, blastocyst-stage embryo, cleavage-stage embryo, fertilization *in vitro*

## Abstract

Older patients or patients with a reduced ovarian response have a low number of embryos, which limits the opportunity for embryo selection. However, for young patients undergoing frozen-thawed embryo transfer (ET), it remains unclear whether embryo stage affects pregnancy outcomes. In the present study, a total of 2952 patients undergoing their first frozen-thawed ET were divided into two groups: patients who had experienced one failed fresh ET (Group A) and patients who had not received fresh ET because of the high risk of ovarian hyperstimulation syndrome (OHSS) (Group B). Our results show that Group B patients had a significantly higher clinical pregnancy rate (CPR) and live birth rate (LBR) than Group A patients. However, Group A patients who underwent blastocyst-stage frozen-thawed ET had a significantly higher CPR and LBR and a lower ectopic pregnancy rate (ePR) than did those who underwent cleavage-stage frozen-thawed ET. In Group B, CPR, ePR, LBR and spontaneous abortion rate (sAR) were similar with blastocyst-stage and cleavage-stage frozen-thawed ET. These results suggest that blastocyst-stage frozen-thawed ET is more appropriate for young patients who had previously undergone one failed fresh ET cycle.

## INTRODUCTION

Frozen-thawed embryo transfer (ET) has been an essential part of assisted reproductive therapies (ART) since the first successful frozen-thawed ET was reported [1]. Frozen-thawed ET enables the redundant embryos generated by *in vitro* fertilization/intra-cytoplasmic sperm injection (IVF/ICSI) to be stored and utilized after one cycle of ovarian stimulation and thus increases cumulative pregnancy rates and reduces the economic burden and physical injury to ART patients. Using freeze-all strategy, frozen-thawed ET can decrease the risk of ovarian hyperstimulation syndrome (OHSS) [2]. It has been shown that ovarian stimulation with gonadotropins impairs endometrial receptivity during fresh ET. Therefore, compared with fresh ET, frozen-thawed ET provides better interaction between embryo/blastocyst and endometrium which leads to a higher clinical pregnancy rate (CPR) [3–6].

To date, for frozen-thawed ET, the selection of cleavage-stage embryo versus blastocyst-stage embryo remains controversial. Although cleavage-stage ET is associated with the generation of additional embryos, morphologically normal cleavage-stage embryos may be chromosomally abnormal or mosaic, leading to higher rates of implantation failure and miscarriage [7]. Blastocyst-stage ET produces fewer embryos for freezing [8], but has the advantages of self-selection and better development potential for the normal embryos [9]. Live birth rate (LBR) has been reported to be significantly higher for patients undergoing fresh blastocyst-stage ET than for those undergoing fresh cleavage-stage ET [10]. However, a recent systematic review and meta-analysis reported no superiority of blastocyst-stage ET over cleavage-stage ET in clinical practice [11]. Especially for young patients with OHSS risk, all of the embryos are typically frozen to prevent the occurrence of OHSS. Thus, to explore the impact of different embryo stages on the pregnancy outcomes will be critical and important to improve the success of frozen-thawed ET.

In this study, we compared the pregnancy outcomes between patients who had experienced one failed fresh ET and those who had not undergone fresh ET because of risk of OHSS. In addition, we evaluated which stage of embryo should be chosen for these two groups of patients.

## RESULTS

### Patients with previous failed fresh ET had lower CPR and LBR for frozen-thawed ET than ET-naïve patients

Group A patients had experienced one failed fresh ET, while Group B patients had not. The general characteristics and pregnancy outcomes of the two groups were presented in Table 1. No significant differences were observed in the age, duration of infertility, BMI, and endometrial thickness on the day of ET. As expected, the antral follicle count (AFC) in Group B was significantly higher than that in Group A (A: 13.58±5.48 vs. B: 17.18±5.78, *p*<0.001). Patients in Group A had a similar proportion of natural cycles and artificial cycles (40.76% vs. 59.25%), while patients in Group B had a greater percentage of artificial cycles (25.74% vs. 74.26%). In addition, both groups had a higher number of cleavage-stage than blastocyst-stage ET (A: 83.67% vs. 16.33%, *p*<0.001; B: 72.69% vs. 27.31%, *p*<0.001). The blastomere survival rate was higher in Group A than in Group B (A: 95.14% vs. B: 93.72%, *p*<0.001). Importantly, although the number of transferred embryos was lower in Group B, the CPR (A: 45.46% vs. B: 49.21%, *p*=0.046) and LBR (A: 37.38% vs. B: 41.27%, *p*=0.034) were significantly higher than in Group A. No significant differences in ectopic pregnancy rate (ePR) or spontaneous abortion rate (sAR) were found between the two groups.

**Table 1:** General characteristics and pregnancy outcomes of Group A (had experienced one failed fresh ET) and Group B (had not received fresh ET because of the high risk of OHSS).

**Variables**	**Group A**	**Group B**	***p* value**
Patient number	1806	1146	
Female age (y)	29.54±2.86	29.41±2.96	0.42
Duration of infertility (y)	3.91±2.48	4.00±2.49	0.34
BMI (kg/m^2^)	21.46±1.85	21.47±1.89	0.42
No. of AFC	13.58±5.48	17.18±5.78	<0.001
Endometrial preparation protocol			
Natural cycle (%)	40.76 (736/1806)	25.74 (295/1146)	<0.001
Artificial cycle (%)	59.25 (1070/1806)	74.26 (851/1146)	
Endometrial thickness on ET day (mm)	10.49±1.96	10.36±1.94	0.76
Type of embryos			
Cleavage-stage embryo (%)	83.67 (1511/1806)	72.69 (833/1146)	<0.001
Blastocyst-stage embryo (%)	16.33 (295/1806)	27.31 (313/1146)	
Blastomere survival rate (%)	95.14 (24637/25895)	93.72 (12255/13076)	<0.001
No. of transferred embryos	2.24±0.67	1.92±0.38	<0.001
CPR (%)	45.46 (821/1806)	49.21 (564/1146)	0.046
ePR (%)	4.14 (34/821)	2.30 (13/564)	0.064
sAR (%)	13.64 (112/821)	13.83 (78/564)	0.92
LBR (%)	37.38 (675/1806)	41.27 (473/1146)	0.034

Data are presented as mean ± standard deviation or percentage (number).

CPR: clinical pregnancy rate; ePR: ectopic pregnancy rate; sAR: spontaneous abortion rate; LBR: live birth rate.

### Patients with previous failed fresh ET had better pregnancy outcomes after blastocyst-stage than cleavage-stage frozen-thawed ET

The general characteristics and pregnancy outcomes of patients in Group A were presented in Table 2. Although more cleavage-stage embryos were transferred than blastocyst-stage embryos (2.34±0.65 vs. 1.69±0.46, *p*<0.001), CPR and LBR were significantly lower (43.28% vs. 56.61%, *p*<0.001; 35.74% vs. 45.76%, *p*=0.001) in patients with cleavage-stage frozen-thawed ET than in those with blastocyst-stage frozen-thawed ET. Importantly, ePR was higher in patients with cleavage-stage frozen-thawed ET than in those with blastocyst-stage frozen-thawed ET (4.89% vs. 1.20%, *p*=0.032). The embryo stages did not significantly affect the sAR (12.54% vs. 17.96%, *p*=0.068). These results demonstrate that patients who had experienced one failed fresh ET had better pregnancy outcomes if they were treated with blastocyst-stage than cleavage-stage frozen-thawed ET.

**Table 2:** General characteristics and pregnancy outcomes of Group A (had experienced one failed fresh ET) and Group B (had not received fresh ET because of the high risk of OHSS) with different transferred embryo-stages.

**Variables**	**Group A**		***p* value**	**Group B**		***p* value**
	**Cleavage-stage**	**Blastocyst-stage**		**Cleavage-stage**	**Blastocyst-stage**	
Patient number	1511	295		833	313	
Female age (y)	29.57±2.86	29.39±2.89	0.45	29.42±2.93	29.39±3.00	0.52
Duration of infertility (y)	3.91±2.47	3.93±2.54	0.91	4.03±2.54	3.89±2.37	0.25
BMI (kg/m^2^)	21.47±1.86	21.39±1.86	0.82	21.47±1.89	21.48±1.86	0.58
No. of AFC	13.45±5.47	14.22±5.52	0.48	16.88±5.79	17.96±5.70	0.86
Endometrial preparation protocol						
Natural cycle (%)	40.37 (610/1511)	42.71 (126/295)	0.45	25.93 (216/833)	25.24 (79/313)	0.81
Artificial cycle (%)	59.63 (901/1511)	57.29 (169/295)	0.45	74.07 (617/833)	74.76 (234/313)	0.81
Endometrial thickness on ET day (mm)	10.52±1.98	10.34±1.89	0.35	10.42±1.95	10.18±1.92	0.29
No. of transferred embryos	2.34±0.65	1.69±0.46	<0.001	2.05±0.26	1.62±0.49	<0.001
CPR (%)	43.28 (654/1511)	56.61 (167/295)	<0.001	48.74 (406/833)	50.48 (158/313)	0.60
ePR (%)	4.89 (32/654)	1.20 (2/167)	0.032	2.21 (9/406)	2.53 (4/158)	0.82
sAR (%)	12.54 (82/654)	17.96 (30/167)	0.068	13.79 (56/406)	13.92 (22/158)	0.97
LBR (%)	35.74 (540/1511)	45.76 (135/295)	0.001	40.93 (341/833)	42.17 (132/313)	0.71

Data are presented as mean ± standard deviation or percentage (number).

### ET-naïve patients had similar pregnancy outcomes between blastocyst-stage and cleavage-stage frozen-thawed ET

The general characteristics and pregnancy outcomes of Group B patients who, due to risk of OHSS, did not experience one failed fresh ET were presented in Table 2. Similar to patients in Group A, a greater number of cleavage-stage embryos were transferred than blastocyst-stage embryos (2.05±0.26 vs. 1.62±0.49, *p<0.001*). However, CPR, ePR, sAR and LBR were similar in patients who received cleavage-stage and blastocyst-stage frozen-thawed ET.

### Blastocyst-stage provides better frozen-thawed ET outcomes than cleavage-stage regardless of endometrial preparation

Since the endometrial preparation protocols may affect the pregnancy results of frozen-thawed ET, we divided the patients into subgroups according to the endometrial preparation protocol (Table 3). Significantly higher CPR was observed for Group A patients who underwent blastocyst-stage frozen-thawed ET than for those who underwent cleavage-stage frozen-thawed ET in both the natural cycle group (58.73% vs.45.57%, *p*=0.007) and the artificial cycle group (55.03% vs. 41.73%, *p*=0.001). Similarly, significantly higher LBR was also observed for Group A patients who underwent blastocyst-stage frozen-thawed ET than for those who underwent cleavage-stage frozen-thawed ET in both the natural cycle group (49.20% vs. 38.69%, *p*=0.029) and the artificial cycle group (42.6% vs. 33.74%, *p*=0.018). In Group B, different stages of frozen-thawed ET did affect the CPR and LBR in both the natural cycle group and the artificial cycle group. Interestingly, although no statistical significances were observed, Group A and Group B patients treated with natural cycle endometrial preparation had higher CPR and LBR than those treated with artificial cycle endometrial preparation regardless of the stage of frozen-thawed ET.

**Table 3:** Comparison of CPR and LBR in Group A (had experienced one failed fresh ET) and Group B (had not received fresh ET because of the high risk of OHSS) with different transferred embryo-stages and endometrial preparation protocols.

**Group**	**Endometrial preparation protocol**	**CPR**				**LBR**		
		**Cleavage-stage**	**Blastocyst-stage**	***p* value**		**Cleavage-stage**	**Blastocyst-stage**	***p* value**
A	Natural cycle	45.57 (278/610)	58.73 (74/126)	0.007		38.69 (236/610)	49.20 (62/126)	0.029
	Artificial cycle	41.73 (376/901)	55.03 (93/169)	0.001		33.74 (304/901)	43.20 (73/169)	0.018
	*p* value	0.14	0.52			0.049	0.31	
B	Natural cycle	51.39 (111/216)	56.96 (45/79)	0.40		44.44 (96/216)	50.63 (40/79)	0.35
	Artificial cycle	47.81 (295/617)	48.29 (113/234)	0.90		39.71 (245/617)	39.32 (92/234)	0.92
	*p* value	0.37	0.18			0.22	0.078	

Data are presented as percentage (number).

## DISCUSSION

Aging is a key factor that affects the ovarian response and pregnancy outcomes for patients with ART treatment. Patients over 35 years old or patients with poor ovarian responses have a limited opportunity for embryo selection because of the limited number of embryos that can be used. Thus, in this study, we specifically selected young patients (≤35 years old) who had sufficient embryos for procedural optimization during IVF treatment. We observed that young patients undergoing frozen-thawed ET who had one previous failed fresh ET (Group A) had lower CPR and LBR than those who had not experienced one failed fresh ET because of the high risk of OHSS (Group B). The lower CPR and LBR may be a consequence of reduced endometrium receptivity or embryo quality in patients who had one failed fresh ET [12]. Endometrium thicknesses were similar on ET day between Group A and Group B patients. It is known that other clinical indexes such as endometrial volume and vascularization index as well as expression levels of endometrial proliferation-related genes have also been used to evaluate the endometrium receptivity [13–17]. Whether other indexes for the endometrium receptivity differ between Group A and Group B patients is unclear and will be an interesting topic for further study. It is worthy to note that, compared to Group A, a higher percentage of Group B patients received artificial cycle endometrial preparation than natural cycle endometrial preparation. Given artificial cycle endometrial preparation did not result in a better pregnancy outcome than natural cycle endometrial preparation, we do not think this factor contributed to the higher CPR and LBR in Group B. Our results also showed that a larger number of transferred embryos did not lead to a higher CPR or LBR in Group A patients indicating that embryo quality may be an important factor to affect the pregnancy outcomes. Notably, although blastomere survival rates were higher in Group A than in Group B patients, the values were close and higher than 90% in both groups. Therefore, we do not anticipate this difference had significant impact on the pregnancy outcomes.

Our results further indicated that blastocyst-stage embryo had higher potential for implantation and growth, which is in accordance with previous studies [9, 18, 19]. Reduced ePR occurrence of blastocyst-stage frozen-thawed ET found in our study is supported by previous studies evaluating the risk of ePR after cleavage-stage ET and blastocyst-stage ET [20, 21]. This is because, compared to the blastocyst-stage embryos, the cleavage-stage embryos usually do not implant immediately and have a higher chance to move back into the fallopian tube via the retrograde contractions of the uterine muscular layer which increases the incidence of the ectopic implantation [22]. Thus, in order to improve hospital-average outcomes, blastocyst-stage frozen-thawed ET is advised for patients with previous failed fresh ET.

Interestingly, CPR, ePR, sAR and LBR were similar in between blastocyst-stage and cleavage-stage frozen-thawed ET in patients who had not experienced one failed fresh ET because of OHSS risk (Group B). We do not know the exact causes of these results. It has been shown that OHSS patients have aberrant levels of hormones and cytokines which can significantly affect the pregnancy outcomes [23]. In addition, although previous studies have demonstrated that blastocyst-stage embryos had higher potential for implantation and growth than cleavage-stage embryos, some disadvantages to blastocyst-stage ET have also been reported [9, 18, 19, 24]. The *in vitro* environment is inferior to the *in vivo* environment, and this difference may lead to the failure of some embryos to blastulate in culture, which could have successfully been implanted if transferred at the cleavage-stage [24]. Moreover, the incidence of transfer cancellation increased, due to a lower number of remaining embryos. Considering the advantages and disadvantages of culturing blastocyst-stage embryos and on the basis of our results, for patients who had not undergone failed fresh ET, blastocyst-stage and cleavage-stage ET can be chosen interchangeably. In clinical application, it is still advisable to look into the need and individual circumstances of each patient.

Whether different endometrial preparation protocols affect the pregnancy outcome remains controversial. Several studies show that the natural cycle endometrial preparation increases implantation rates, especially when transferring blastocysts [25, 26]. However, other studies report no difference in CPR or LBR among the different endometrial preparation protocols [27, 28]. In the present study, although no statistical significances were observed, Group A and Group B patients treated with natural cycle endometrial preparation had higher CPR and LBR for frozen-thawed ET than those treated with artificial cycle endometrial preparation regardless of the embryonic stage. Therefore, future study with large patient number is needed to examine the effect of different endometrial preparation protocols on the pregnancy outcome for the patients with frozen-thawed ET.

In summary, our results show that blastocyst-stage frozen-thawed ET may be recommended for young patients with a previous failed fresh ET. However, for patients who had not undergone fresh ET because of the high risk of OHSS, blastocyst-stage or cleavage-stage frozen-thawed ET can be chosen interchangeably. Our study provides an actionable recommendation for the transfer stage of embryo for the patients with frozen-thawed ET to achieve better hospital-average success rate.

## MATERIALS AND METHODS

### Patients

This retrospective study included all frozen-thawed ET cycles from the Center for Reproductive Medicine of the First Affiliated Hospital of Zhengzhou University from January 2014 to December 2017. Informed consent was obtained from all patients. The study received approval and was carried out in accordance with the approved guidelines from the Zhengzhou University Research Ethics Board.

**Figure 1:**
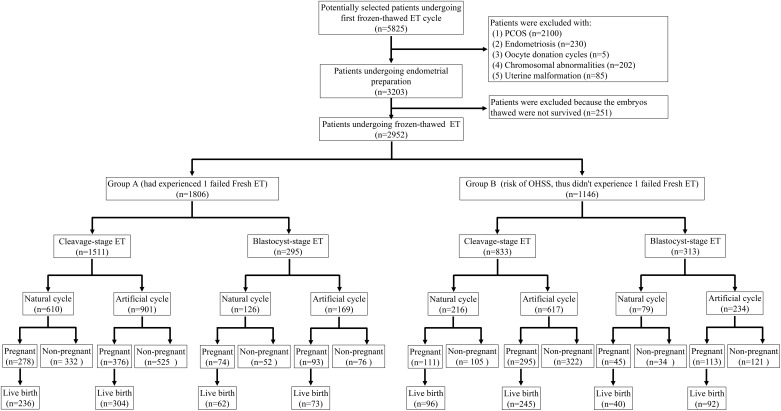
**Flow chart of patient selection.**

A total of 5825 patients were selected for this study and 2952 were enrolled in the final analysis (Figure 1). Young patients with normal ovarian reserves undergoing the first frozen-thawed ET were included. The inclusion criteria were as follows: 1) age: ≤35 years old; 2) body mass index (BMI): 18-25 kg/m^2^; 3) FSH: <10 mIU/mL; 4) AFC: >6; and 5) cause of infertility: tubal pathology, male factors and unexplained factors. The exclusion criteria were: 1) polycystic ovarian syndrome (PCOS); 2) endometriosis; 3) oocyte donation cycles; 4) parent chromosomal abnormalities; 5) uterine malformation; and 6) thawed embryos did not survive.

The 2952 patients were divided into Group A (had experienced one failed fresh ET) and Group B (risk of OHSS, thus did not experience one failed fresh ET). Group A definition: For patients without OHSS risk, one or two embryos of the highest quality selected by the embryologist were transferred to the uterine cavity on the third or fifth day after oocyte retrieval, and the remaining embryos/blastocysts were frozen. If these patients did not become pregnant, then they were enrolled for their first frozen-thawed ET. Group B definition: For patients with a high risk of OHSS, all embryos/blastocysts were frozen in the fresh ET cycle. The diagnostic criteria for patients who did not undergo fresh ET because of the high risk of OHSS were as follows: 1) serum estradiol ≥5000 pg/mL on the day of hCG administration; 2) more than 15 oocytes retrieved; 3) ovarian diameter longer than 8 cm; 4) ascitic or pleural fluid detected by ultrasound; or 5) symptoms, such as abdominal distention and chest distress. OHSS risk was identified if the patients had one of the above symptoms. These patients were enrolled for their first frozen-thawed ET.

Sample size estimation: The CPR of Group A (~43.18%) and Group B (~46.52%) in our center was considered when calculating the sample size. The ratio of the sample size of Group A to that of Group B was 1.5, suggesting that to detect a significant difference in CPR between Group A and B with α=0.05 and β=0.10, at least 1785 cycles in Group A and 1071 cycles in Group B are needed. We therefore terminated the study when the CPR comparison between Group A and B showed significant differences (p≤0.05), and at this end point, 1806 cycles and 1146 cycles were included in Group A and Group B, respectively.

### Controlled ovarian hyperstimulation protocol

In fresh ET, all patients were treated with the standard long protocol. Pituitary was suppressed with sc administration of 3.75 mg triptorelin acetate (Ipsen Pharma Biotech, France). When the patient achieved the criteria for pituitary suppression, ovarian stimulation was initiated with gonadotropin (Gonal-F, Merck, Germany; Puregon, Organon, Netherlands; Urofollitropin, Livzon, China). The gonadotropin dose range was 75-300 IU based on the ovarian response (75-150 IU for normal or high ovarian response; 150-300 IU for reduced ovarian response). Exact dose of gonadotropin was adjusted to general and clinical characteristics of individual patient. When at least three follicles had reached 18 mm and the lead follicle was ≥20 mm, hCG (Livzon) was injected to trigger oocyte maturation. Oocyte retrieval was scheduled at 36 h after hCG injection by transvaginal ultrasound-guided follicular aspiration. Progesterone in oil was used for luteal support at a dose of 60 mg per day after oocyte pick-up.

### Embryo/blastocyst vitrification and warming

The embryo/blastocyst vitrification and warming protocols were followed according to the system used in the Reproductive Medicine Center of the First Affiliated Hospital of Zhengzhou University [29]. During the fresh ET, three embryos of good quality were chosen by the embryologist and frozen at the third day; the remaining embryos were cultured to blastocyst stage and then frozen. Clinician defined the embryo stage for transfer and freeze. The selection of cleavage-stage or blastocyst-stage embryos for ET depended on the availability of blastocyst-stage embryos, suggestion from clinician and patients’ choice. The embryos were defined as viable when more than 50% of the blastomeres survived. The blastocysts were regarded as viable when more than half of the cells were intact and the blastocoele was expanded.

### Endometrial preparation

The endometrial preparation protocol for frozen-thawed ET included natural cycles and artificial cycles [29]. Patients with regular menstrual cycles were treated with natural cycle, while patients with irregular menstrual cycles were treated with artificial cycle. For the natural cycles, cleavage-stage and blastocyst-stage frozen-thawed ET were performed at 4 and 6 days after ovulation, respectively. Ovulation was monitored by serum LH levels and transvaginal ultrasound. Ovulation usually occurred 36-40 h after the rise in serum LH levels, which was also confirmed by the dominant follicular rupture observed by the transvaginal ultrasound. For the artificial cycles, 2-4 mg of estradiol was given between days 2 and 4 of the menstrual cycle. The estradiol treatment was continued and dose was adjusted according to the endometrial thickness measured by transvaginal ultrasound. When the endometrial thickness was observed to be over 7 mm with a triple-line appearance, patients began a daily intramuscular injection of 60 mg progesterone. Cleavage-stage and blastocyst-stage frozen-thawed ET were initiated at 5 and 7 days after the progesterone injection, respectively. Progesterone was administered until the pregnancy test was performed. If the pregnancy test was positive, progesterone supplementation was sustained for another 12 weeks.

### Pregnancy results evaluation

The following pregnancy results were evaluated: clinical pregnancy rate (CPR), ectopic pregnancy rate (ePR), spontaneous abortion rate (sAR), and live birth rate (LBR). Biochemical pregnancy was diagnosed according to an increase in the serum β-hCG concentration at 14 days after ET. CPR was determined by the identification of a gestational sac by abdominal ultrasound at 35 days after ET. ePR was defined as the number of ectopic pregnancies divided by the number of clinical pregnancies. sAR was defined as a pregnancy loss following sonographic visualization of an intrauterine gestational sac at 5-6 weeks of gestation. LBR was defined as the birth of a healthy child.

### Statistical analysis

The data are presented as the mean ± standard deviation or percentage (number). The data were analyzed using SPSS version 19.0 (SPSS, Chicago, IL, USA). ANOVA, t-test, χ^2^ test, and Fisher’s exact test were used when appropriate. The significance level was set at *p*<0.05.
